# Supplementary material for: Unveiling the Bioactive Potential of the Invasive Jellyfish Phyllorhiza punctata Through Integrative Transcriptomic and Proteomic Analyses
Source: Biomolecules. 2025 Aug 4;15(8):1121. doi: 10.3390/biom15081121 (PMC12383608; doi:10.3390/biom15081121)
Supplement: Supplementary file 1 [file biomolecules-15-01121-s001.zip › Suplementary Material/FileS3/PEPFOLD4/XP_065061945_15770/clusters.html]

 PEP-FOLD clusters


Download archive of all models  here   

|  |  |  |
| --- | --- | --- |
| # | file | sOPEP |
| # cluster 1 - Size: 98 |  |  |
|  | XP\_065061945\_15770-model1 | -21.3175 |
|  | XP\_065061945\_15770-model1.1 | -21.2908 |
|  | XP\_065061945\_15770-model1.2 | -21.2908 |
|  | XP\_065061945\_15770-model1.3 | -21.2854 |
|  | XP\_065061945\_15770-model1.4 | -21.208 |
|  | XP\_065061945\_15770-model1.5 | -21.208 |
|  | XP\_065061945\_15770-model1.6 | -21.2037 |
|  | XP\_065061945\_15770-model1.7 | -21.2018 |
|  | XP\_065061945\_15770-model1.8 | -21.1994 |
|  | XP\_065061945\_15770-model1.9 | -21.1758 |
|  | XP\_065061945\_15770-model1.10 | -21.1511 |
|  | XP\_065061945\_15770-model1.11 | -21.1511 |
|  | XP\_065061945\_15770-model1.12 | -21.1388 |
|  | XP\_065061945\_15770-model1.13 | -21.129 |
|  | XP\_065061945\_15770-model1.14 | -21.0848 |
|  | XP\_065061945\_15770-model1.15 | -21.0848 |
|  | XP\_065061945\_15770-model1.16 | -21.0822 |
|  | XP\_065061945\_15770-model1.17 | -21.0822 |
|  | XP\_065061945\_15770-model1.18 | -21.0773 |
|  | XP\_065061945\_15770-model1.19 | -21.0674 |
|  | XP\_065061945\_15770-model1.20 | -21.0605 |
|  | XP\_065061945\_15770-model1.21 | -21.0579 |
|  | XP\_065061945\_15770-model1.22 | -21.0563 |
|  | XP\_065061945\_15770-model1.23 | -21.0563 |
|  | XP\_065061945\_15770-model1.24 | -21.0563 |
|  | XP\_065061945\_15770-model1.25 | -21.0517 |
|  | XP\_065061945\_15770-model1.26 | -21.0502 |
|  | XP\_065061945\_15770-model1.27 | -21.0437 |
|  | XP\_065061945\_15770-model1.28 | -21.0437 |
|  | XP\_065061945\_15770-model1.29 | -21.0436 |
|  | XP\_065061945\_15770-model1.30 | -21.0267 |
|  | XP\_065061945\_15770-model1.31 | -21.0267 |
|  | XP\_065061945\_15770-model1.32 | -21.0235 |
|  | XP\_065061945\_15770-model1.33 | -21.0224 |
|  | XP\_065061945\_15770-model1.34 | -21.0173 |
|  | XP\_065061945\_15770-model1.35 | -21.0167 |
|  | XP\_065061945\_15770-model1.36 | -21.0167 |
|  | XP\_065061945\_15770-model1.37 | -21.01 |
|  | XP\_065061945\_15770-model1.38 | -21.0093 |
|  | XP\_065061945\_15770-model1.39 | -21.0072 |
|  | XP\_065061945\_15770-model1.40 | -20.9917 |
|  | XP\_065061945\_15770-model1.41 | -20.9871 |
|  | XP\_065061945\_15770-model1.42 | -20.9871 |
|  | XP\_065061945\_15770-model1.43 | -20.9871 |
|  | XP\_065061945\_15770-model1.44 | -20.9747 |
|  | XP\_065061945\_15770-model1.45 | -20.9696 |
|  | XP\_065061945\_15770-model1.46 | -20.9685 |
|  | XP\_065061945\_15770-model1.47 | -20.965 |
|  | XP\_065061945\_15770-model1.48 | -20.965 |
|  | XP\_065061945\_15770-model1.49 | -20.9616 |
|  | XP\_065061945\_15770-model1.50 | -20.9616 |
|  | XP\_065061945\_15770-model1.51 | -20.9603 |
|  | XP\_065061945\_15770-model1.52 | -20.9578 |
|  | XP\_065061945\_15770-model1.53 | -20.947 |
|  | XP\_065061945\_15770-model1.54 | -20.9466 |
|  | XP\_065061945\_15770-model1.55 | -20.9426 |
|  | XP\_065061945\_15770-model1.56 | -20.9421 |
|  | XP\_065061945\_15770-model1.57 | -20.9421 |
|  | XP\_065061945\_15770-model1.58 | -20.9368 |
|  | XP\_065061945\_15770-model1.59 | -20.9354 |
|  | XP\_065061945\_15770-model1.60 | -20.9354 |
|  | XP\_065061945\_15770-model1.61 | -20.929 |
|  | XP\_065061945\_15770-model1.62 | -20.9234 |
|  | XP\_065061945\_15770-model1.63 | -20.9234 |
|  | XP\_065061945\_15770-model1.64 | -20.9234 |
|  | XP\_065061945\_15770-model1.65 | -20.9233 |
|  | XP\_065061945\_15770-model1.66 | -20.9225 |
|  | XP\_065061945\_15770-model1.67 | -20.9225 |
|  | XP\_065061945\_15770-model1.68 | -20.9198 |
|  | XP\_065061945\_15770-model1.69 | -20.9191 |
|  | XP\_065061945\_15770-model1.70 | -20.9191 |
|  | XP\_065061945\_15770-model1.71 | -20.9183 |
|  | XP\_065061945\_15770-model1.72 | -20.9126 |
|  | XP\_065061945\_15770-model1.73 | -20.9117 |
|  | XP\_065061945\_15770-model1.74 | -20.9115 |
|  | XP\_065061945\_15770-model1.75 | -20.9104 |
|  | XP\_065061945\_15770-model1.76 | -20.9063 |
|  | XP\_065061945\_15770-model1.77 | -20.9063 |
|  | XP\_065061945\_15770-model1.78 | -20.8957 |
|  | XP\_065061945\_15770-model1.79 | -20.8917 |
|  | XP\_065061945\_15770-model1.80 | -20.8823 |
|  | XP\_065061945\_15770-model1.81 | -20.8814 |
|  | XP\_065061945\_15770-model1.82 | -20.8583 |
|  | XP\_065061945\_15770-model1.83 | -20.8561 |
|  | XP\_065061945\_15770-model1.84 | -20.8508 |
|  | XP\_065061945\_15770-model1.85 | -20.8484 |
|  | XP\_065061945\_15770-model1.86 | -20.8465 |
|  | XP\_065061945\_15770-model1.87 | -20.8342 |
|  | XP\_065061945\_15770-model1.88 | -20.8311 |
|  | XP\_065061945\_15770-model1.89 | -20.8282 |
|  | XP\_065061945\_15770-model1.90 | -20.8115 |
|  | XP\_065061945\_15770-model1.91 | -20.7917 |
|  | XP\_065061945\_15770-model1.92 | -20.7782 |
|  | XP\_065061945\_15770-model1.93 | -20.7691 |
|  | XP\_065061945\_15770-model1.94 | -20.7691 |
|  | XP\_065061945\_15770-model1.95 | -20.7604 |
|  | XP\_065061945\_15770-model1.96 | -20.7465 |
|  | XP\_065061945\_15770-model1.97 | -20.7393 |
| # cluster 2 - Size: 1 |  |  |
|  | XP\_065061945\_15770-model2 | -16.4135 |
| # cluster 3 - Size: 1 |  |  |
|  | XP\_065061945\_15770-model3 | -4.42015 |
|  |  |  |
